# Associations of Physical Activity, Screen Time with Depression, Anxiety and Sleep Quality among Chinese College Freshmen

**DOI:** 10.1371/journal.pone.0100914

**Published:** 2014-06-25

**Authors:** Qi Feng, Qing-le Zhang, Yue Du, Yong-ling Ye, Qi-qiang He

**Affiliations:** 1 School of Public Health, Wuhan University, Wuhan, P. R. China; 2 Global Health Institute, Wuhan University, Wuhan, P. R. China; Institute of Psychiatry, United Kingdom

## Abstract

**Objectives:**

To investigate the independent and interactive associations of physical activity (PA) and screen time (ST) with depression, anxiety and sleep quality among Chinese college students.

**Methods:**

A cross-sectional study was conducted in Wuhan University, China from November to December 2011. The students reported their PA, ST and socio-economic characteristics using self-administered questionnaires. Sleep quality was measured by the Pittsburgh Sleep Quality Index (PSQI). Depression and anxiety were assessed using the Self-rating Depression Scale (SDS) and Self-rating Anxiety Scale (SAS), respectively. Multivariate logistic regression models were used to estimate the odds ratios (ORs) and 95% confidence intervals (CIs) of the independent and interactive relationships of PA and ST with depression, anxiety and sleep quality.

**Results:**

A total of 1106 freshmen (471 females and 635 males) aged 18.9±0.9 years were included in the study. After adjustment for potential confounders, high PA and low ST were independently associated with significantly lower risks for poor sleep quality (OR: 0.48, 95% CI: 0.30–0.78) and depression (OR: 0.67, 95%CI: 0.44–0.89), respectively. An interactive inverse association was observed for combined effects of PA and low ST on depression (OR: 0.62, 95%CI: 0.40–0.92) and sleep quality (OR: 0.51, 95%CI: 0.27–0.91). No statistically significant associations were found between PA, ST and anxiety among the participants.

**Conclusions:**

These findings suggest an independent and interactive relationship of high PA and low ST with significantly reduced prevalence of depressive problems and favorable sleep quality among Chinese college freshmen.

## Introduction

College students are special populations that are enduring a period of great challenges, risks and social developmental transition. Previous studies have shown high rates of mental disorders (typically depression and anxiety) among college students around the world [1], [2]. Besides, a high prevalence of poor sleep quality was found in this population [3], [4]. Insufficient sleep is associated with poor mental health, quality of life, and academic performance [5], [6]. Thus, promoting sleep quality and preventing mental disorders may have broad benefits in this population.

Although there are abundant studies showing favorable effects of physical activity (PA) on mental health and sleep quality [7], [8], the collective evidence continues to emerge and remains inconclusive. Some studies have failed to find an association between PA and sleep quality [9], [10], whereas being active showed no beneficial impacts on mental health [11]. Inconsistent results might be caused by the heterogeneity of study samples, exposure and outcome assessment, and various confounders.

Previous researchers have pointed out that investigations of PA without consideration of sedentary behaviors may have resulted in an overstated assumption of health effects [12]. Overall, sedentary behaviors in college students are largely due to increased screen time (ST), mainly including computer and internet usage [13]. ST has been associated with obesity [14], unfavorable blood lipids [15], backache and headache [16], and poor school performance [17] in children and adolescents. The American Academy of Pediatrics recommends no more than 2 h/day of ST in children and adolescents [18]. However, the interaction effects of PA and ST on mental health and sleep quality among college students have been little studied. Therefore, we conducted a cross-sectional study to investigate the independent and interactive associations of PA and ST with depression, anxiety and sleep quality among Chinese college freshmen.

## Methods

### Sample and data collection

This study was conducted in Wuhan University, a large public university located in central China, from November to December 2011. The primary objective of this study was to investigate the association between PA and mental health in Chinese college freshmen. Assuming α = 0.05 and the power of the study (1-β)  = 0.80, 978 participants would be needed to detect a difference of 2 standard Self-rating Depression Scale (SDS) and Self-rating Anxiety Scale (SAS) scores between high PA and low PA group [19], [20]. To account for possible incomplete or non-returned questionnaires, we aimed to sample an extra 10%, which resulted in 1077 students. Participants were recruited by a two-stage random cluster method. Step 1, two schools were randomly selected from each faculty (Humanities, social sciences, sciences, engineering, information sciences, and medical sciences) of Wuhan University. Step 2, Two classes in grade 1 (around 50 students each class) from each school were selected using a simple random sampling method. All students were enrolled into the survey.

The survey was performed in the classrooms by trained investigators using self-administered questionnaires. The investigators provided support to the participants and maintained order in the classrooms. The questionnaire contained information on the students' age, height, weight, socio-demographic, PA, ST (defined as time spent on computer including internet use, watching TV/video program and playing games), sleep quality, depression and anxiety status (Questionnaire S1, S2). Written informed consent was obtained from all students who participated in the study. The study was approved by the Medical Research Ethics Committee of Wuhan University.

### Measures

The frequency of PA (How often do you sport and/or vigorous free play each week with 30 minutes at least per day?) was assessed by using the following options: daily, 5–6 days/week, 3–4 days/week, 1–2 days/week, and less than once per week. Subjects with high PA were defined as those who sported and/or vigorous free played at least three days per week[21]. The subjects also reported their ST (How many hours per day do you spend on computer, including internet use, watching TV/video programs and playing games on a usual weekday and weekend day, respectively?). An average of hours of ST (weekdays and weekend days) was calculated to obtain an overall average ST. The present study categorized ST as ≤2 hours/day (low ST) and >2 hours/day (high ST).

Sleep quality was measured by the Pittsburgh Sleep Quality Index (PSQI) [22]. The PSQI is a 19-item self-reported questionnaire that evaluates sleep quality over the past month, which yields seven component scores: sleep latency, duration of sleep, habitual sleep efficiency, sleeps disturbances, use of sleep medications, daytime dysfunction, and overall sleep quality. The sum of scores for these seven components yields the PSQI global score, with higher scores indicating poor sleep quality during the previous month. Based on the prior literature, PSQI global scores >5 were defined as “poor sleep quality” [23].

Depression and anxiety were assessed using the SDS and SAS, respectively [24], [25]. These scales are standard assessment instruments, and their reliability and validity have been examined in Chinese population [26]–[28]. Higher scores on the SDS or SAS indicated a higher level of mental disorder. According to the Chinese norm for the SDS and SAS which reflects the subjective feelings of subjects with anxiety tendencies or depression severity, a total standard score of 53 or 50 was set as a cut-off point of depression or anxiety, respectively [29].

### Statistical analysis

Students were included in the present study if they aged between 16 to 24 years of age and did not have any chronic disease. Respondents who had more than 10% items with missing data were excluded. Otherwise, the missing data were handled in two ways: for categorical variables, the missing value was recoded as a dummy variable; for continuous variables, the missing item was replaced by the mean value. The differences in the characteristics between males and females were determined using t-test and Chi-square test, where appropriate. Multivariate logistic regression models were used to estimate the odds ratios (ORs) and 95% confidence intervals (CIs) of the independent and interactive relationships of PA and ST with depression, anxiety and sleep quality, after adjusting for sex, age, body mass index (BMI, kg/m^2^), and maternal education. Statistical analyses were performed using the SPSS statistical package (version 13.0; SPSS Inc, Chicago, Ill. USA).

## Results

Of a total of 1200 participants, 1106 (92.2%) who met the inclusion criteria were included in the final data-analysis. The study subject's characteristics stratified by gender are shown in Table 1. 471 students (42.6%) were females, the age of participants ranged from 16–24 years, with a mean age of 18.9 (SD = 0.9). A majority of the students' parents had median to high education levels (88.2% in fathers and 74.0% in mothers, respectively). 81.6% students reported low PA while 72.7% had ST >2 h/day. The prevalence of poor sleep quality, depression and anxiety was 17.7%, 10.6%, and 7.6%, respectively. Compared to males, females had significantly lower age, BMI, as well as higher prevalence of low PA. Nevertheless, the differences in the prevalence of poor sleep quality, depression and anxiety between males and females were not statistically different.

**Table 1:** Basic characteristics of the study subjects.

	Total (n = 1106)	Male(n = 635)	Female (n = 471)	p value
Age, years	19.0 (0.9)	19.0(1.0)	18.8(0.9)	<0.001
BMI, kg/m^2^	20.4 (2.4)	20.7(2.5)	19.9(2.3)	<0.001
Paternal education				0.007
Low (≤6 years)	131 (11.8)	77(12.1)	54(11.5)	
Median (7–12 years)	632 (57.2)	387(60.9)	245(52.0)	
High (>12 years)	343 (31.0)	171(26.9)	172(36.5)	
Maternal education				0.006
Low (≤6 years)	289 (26.1)	183 (28.7)	106 (22.5)	
Median (7–12 years)	569 (51.5)	330 (52.0)	239 (50.7)	
High (>12 years)	248 (22.5)	122 (19.2)	126 (26.8)	
PA				<0.001
Low	902 (81.6)	497(78.3)	405(86.0)	
High	204 (18.4)	138(21.7)	66(14.0)	
ST level				0.195
>2 h/day	804 (72.7)	452(71.2)	352(74.7)	
≤2 h/day	302 (27.3)	183(28.8)	119(25.3)	
Sleep quality-poor	196 (17.7)	111(17.5)	85(18.0)	0.812
Depression	117 (10.6)	65(10.2)	52(11.0)	0.693
Anxiety	84 (7.6)	43(6.8)	41(8.7)	0.252

Values are presented as mean (SD) or number (percentage) when appropriate.

BMI: body mass index. PA: physical activity. ST: screen time.

Table 2 displays the independent association of PA and ST with depression, anxiety and sleep quality. After adjustment for potential confounders, high PA and low ST were independently associated with significantly lower risks for poor sleep quality (OR: 0.48, 95%CI: 0.30–0.78) and depression (OR: 0.67, 95%CI: 0.44–0.89), respectively. No significant associations were found between PA, ST and anxiety among the participants.

**Table 2:** Associations between physical activity, screen time and depression, anxiety, sleep quality among Chinese college freshmen.

	Depression		Anxiety		Poor sleep quality	
	n (%)	OR(95%CI)	n (%)	OR(95%CI)	n (%)	OR(95%CI)
PA						
Low	100(11.1)	Ref.	74(8.2)	Ref.	273(30.3)	Ref.
High	17(8.3)	0.74(0.43,1.28)	10(4.9)	0.61(0.31,1.20)	41(20.1)	0.48(0.30,0.78)**
ST						
>2 h/day	40(13.2)	Ref.	17(.6)	Ref.	50(16.6)	Ref.
≤2 h/day	77(9.6)	0.67(0.44,0.89)*	67(8.3)	1.52(0.87,2.64)	146(18.2)	1.11(0.77,1.58

Adjusted for age, gender, BMI, and maternal education.

*: p<0.05; **: p<0.01.

OR: odd ratio. CI: confidence interval. PA: physical activity. ST: screen time.

An interactive inverse association (Table 3) was observed for the combined effects of low PA/low ST on depression (OR: 0.62, 95%CI: 0.40–0.92) and high PA/low ST on sleep quality (OR: 0.51, 95%CI: 0.27–0.91), respectively. Compared with those who had low PA and >2 h/day ST levels, subjects with high PA showed statistically insignificant lower risks for anxiety (for >2 h/day: OR: 0.70, 95%CI: 0.19–2.52; for ≤2 h/day: OR: 0.92, 95%CI: 0.36–2.33).

**Table 3:** Multivariate logistic regression for depression, anxiety, and sleep quality by combined physical activity and screen time among Chinese college freshmen.

	ST	Low PA		High PA	
		n (%)	OR(95%CI)	n (%)	OR(95%CI)
Depression	>2 h/day	33(14.5)	Ref.	7(9.5)	0.61(0.26,1.46)
	≤2 h/day	67(9.9)	0.62(0.40,0.92)*	10(7.7)	0.49(0.23,1.04)
Anxiety	>2 h/day	14(6.1)	Ref.	3(4.1)	0.70(0.19,2.52)
	≤2 h/day	60(8.9)	1.51(0.81,2.77)	7(5.4)	0.92(0.36,2.33)
Poor sleep quality	>2 h/day	43(18.9)	Ref.	7(9.5)	0.44(0.19,1.03)
	≤2 h/day	132(19.6)	1.02(0.69,1.51)	14(10.8)	0.51(0.27,0.91)*

Adjusted for age, gender, BMI, and maternal education.

*: p<0.05.

OR: odd ratio. CI: confidence interval. PA: physical activity. ST: screen time.

## Discussion

Findings from the present study indicate that PA and ST were independently associated with self-reported poor sleep quality and depression risks in Chinese college freshmen, respectively. In addition, the interactive analysis showed that, compared to their counterparts, the subjects with high PA and low ST had 49% lower odds of poor sleep quality, whereas within the low PA group, subjects with low ST were 38% less likely to report depression than their peers.

Several previous studies have shown that regular PA can promote mental health in various ways, including improved self-esteem, self-efficacy, cognitive function, psychological function, and decreased distress, etc [7], [30]. Furthermore, exercise levels are positively related to sleep quality in adolescents and young adults [8], [23]. Nevertheless, only a few studies have examined the relationships of combinations of PA and ST with mental health and sleep quality. In a multi-ethnic Asian population, Sloan et al. found physically active adults aged 18–79 years who accumulated 5 h/day or less secondary behaviors had 40% lower odds of psychological distress [12]. Another study on Chinese urban adolescents aged 11–16 years showed that high ST and insufficient vigorous PA interacted to increase psychological problems [31]. Our results not only add to the existing findings showing a favorable independent effect of high PA and low ST among adolescents and young adults, but also extend the current literatures by examining the interactive effects of PA and ST on mental health and sleep quality among college students, who have a higher prevalence of mental health problems than the general population, as well as represent one of the most sleep-deprived population.

In our study, the significantly lower risks for depression in subjects with low ST compared to those high ST were only found within the low PA groups, although high PA was associated with insignificantly reduced risks for depression. The primary objective of this study was to investigate the association between physical activity and mental health in Chinese college freshmen. Therefore, the sample in each sub-group in the interactive analyses was relative small, leading to low power of the present study. Another possible reason for the lack of significant associations among high PA group may be because of the measures of PA used. The self-reported PA may reflect only a small amount of activities. In our study, PA was assessed by asking the participants to recall the frequency of their sports and/or vigorous free play, but not leisure-time PA (e.g. leisure walking, walk to or from class/dormitory room/library, etc.). It is possible that individuals who reported low sport PA had high leisure-time PA. Thus, this might have caused misclassification of PA, which could have weakened the associations. Although a small-to-moderate effect for PA on anxiety has been previously reported [32], the negative associations between PA/ST and anxiety are in line with a systemic review by Larun et al [33]. They found that the exercise interventions only showed a negative effect on anxiety among young people. A meta-analysis of randomized controlled trials also revealed no beneficial effect of exercise interventions on anxiety symptoms [34]. Given the inconclusive results, there is a need for well-designed studies with larger sample size and objective measure of PA and ST to provide more valuable information for researchers.

Although regular PA has been found to improve sleep quality [23], some studies also showed no significant effects of exercise or daily PA on sleep [36], [37]. The results of our investigation lend support to the few previous studies showing a favorable association of PA engagement and reduced sedentary time with sleep quality [23], [35]. There are several possible explanations for this association: 1) PA produces physiological changes, such as greater sleep efficiency and shorter sleep onset latency, which contribute substantially to favorable sleep quality [38]; 2) the benefits of PA on sleep quality are mediated by improving mental health, including decreased symptoms of depression and increased self-esteem [32]; 3) physically active subjects tend to practice several other healthy behaviors, such as favorable dietary habits and less caffeine use which are associated with better sleep quality [39], [40].

Limitations of this study should be recognized when interpreting the results. First, because of a cross-sectional study design of the present study, we cannot infer a causal direction. Second, although the presence of mental health problems and poor sleep quality was assessed by standardized questionnaires, these measures are not equivalent to clinical diagnoses, thus future studies with diagnostic interviews should be used. Third, the PA and ST levels were self-reported; therefore, recall and reporting bias cannot be excluded. Fourth, PA measures did not include its intensity and leisure-time PA. Smart phone use was not included in the definition of ST, and which types of ST may be relevant remain unclear. Finally, the subjects recruited from one large Chinese university were general healthy and well-educated, and one should be cautious in generalizing our findings to Chinese young adults.

In summary, the present study suggests an independent and interactive relationship of high PA and low ST with significantly reduced prevalence of mental health problems and favorable sleep quality among Chinese college freshmen. Our results provide support for the notion that maintaining sufficient PA and reducing sedentary behaviors should be included in the planning of health promotion strategies.

## Supporting Information

Questionnaire S1 **Questionnaire in Chinese.**
(DOC)

Questionnaire S2 **Questionnaire in English.**
(DOC)
