# Supplementary material for: Associations of Physical Activity, Screen Time with Depression, Anxiety and Sleep Quality among Chinese College Freshmen
Source: PLoS One. 2014 Jun 25;9(6):e100914. doi: 10.1371/journal.pone.0100914 (PMC4071010; doi:10.1371/journal.pone.0100914)
Supplement: Questionnaire S1 — Questionnaire in Chinese. (DOC) [file pone.0100914.s001.doc]

**调查问卷**

1. 你的性别？ ①男 ②女

2. 你的出生年月是？ _____年____月___日

3.你的身高 ________厘米，体重________千克

4. 您是否患有高血压、心脏病、哮喘等慢性疾病？

①没有 ②有

5.您父亲的学历是：

①大专及以上(12年以上教育) ②高中、中专或技校（10-12年教育） ③初中（7-9年教育） ④小学及以下（≤6年教育）

6.您母亲的学历是：

①大专及以上(12年以上教育) ②高中、中专或技校（10-12年教育） ③初中（7-9年教育） ④小学及以下（≤6年教育）

7. 你每周进行体育锻炼和/或积极活动的频率如何？（**每天至少30分钟**）?

①每天 ② 5-6天/周 ③ 3-4天/周 ④ 1-2天/周 ⑤ <1天/周

8. **周1到周5**，你平均**每天**用电脑(包括上网/玩游戏/看电视、电影节目)是_____小时。

9. **周6到周日**，你平均**每天**用电脑(包括上网/玩游戏/看电视、电影节目)是 _____小时。

**请从以下每一个问题中选一个最符合您的情况作答，在相应的方框内打“√”。**

10. **最近一个月**您是否因为以下问题而经常睡眠不好：

| **问题** | **过去一个月没有** | **<1个晚上/周** | **1-2个晚上/周** | **≥ 3个晚上/周** |
| --- | --- | --- | --- | --- |
| A入睡困难（不能在30分钟内入睡） |  |  |  |  |
| B夜间易醒或早醒 |  |  |  |  |
| C夜间起床上洗手间 |  |  |  |  |
| D呼吸不畅 |  |  |  |  |
| E大声咳嗽或打鼾声 |  |  |  |  |
| F感觉冷 |  |  |  |  |
| G感觉热 |  |  |  |  |
| H做恶梦 |  |  |  |  |
| I疼痛不适 |  |  |  |  |
| J其他影响睡眠的事情 |  |  |  |  |

11. 最近一个月，总的来说，您认为自己的睡眠质量：

①很好 ②较好 ③较差 ④很差

12. 最近一个月，您是否经常要服药（包括从以医生处方或者在外面药店购买）才能入睡？

①过去一个月没有 ② <1个晚上/周 ③1-2个晚上/周 ④ ≥ 3个晚上/周

13. 最近一个月，您常感到困倦吗？

①过去一个月没有 ② <1个晚上/周 ③1-2个晚上/周 ④ ≥ 3个晚上/周

14. 最近一个月，您感到做事情的精力不足吗？

①没有 ②偶尔有 ③有时有 ④经常有

**I.请仔细阅读以下每个条目，并根据您最近一周的实际感觉，在相应的方框内打钩“√”。**

| **最近一周中，您有无以下感受/情形？** | **没有或很少有** | **有时有** | **大部分时间有** | **绝大部分时间有** |
| --- | --- | --- | --- | --- |
| 1．我感到情绪沮丧，郁闷 |  |  |  |  |
| 2．我感到早晨心情最好 |  |  |  |  |
| 3．我要哭或想哭 |  |  |  |  |
| 4．我夜间睡眠不好 |  |  |  |  |
| 5．我吃饭像平时一样多 |  |  |  |  |
| 6．我的性功能正常 |  |  |  |  |
| 7．我感到体重减轻 |  |  |  |  |
| 8．我为便秘烦恼 |  |  |  |  |
| 9．我的心跳比平时快 |  |  |  |  |
| 10．我无故感到疲劳 |  |  |  |  |
| 11．我的头脑像往常一样清楚 |  |  |  |  |
| 12．我做事情像平时一样不感到困难 |  |  |  |  |
| 13．我坐卧不安，难以保持平静 |  |  |  |  |
| 14．我对未来感到有希望 |  |  |  |  |
| 15．我比平时更容易激怒 |  |  |  |  |
| 16．我觉得决定什么事很容易 |  |  |  |  |
| 17．我感到自己是有用的和不可缺少的人 |  |  |  |  |
| 18．我的生活很有意义 |  |  |  |  |
| 19．假若我死了别人会过得更好 |  |  |  |  |
| 20．我仍旧喜爱自己平时喜爱的东西 |  |  |  |  |
| 21．我觉得比平常容易紧张或着急 |  |  |  |  |
| 22．我无缘无故地感到害怕 |  |  |  |  |
| 23．我容易心里烦乱或觉得惊恐 |  |  |  |  |
| 24．我觉得我可能将要发疯 |  |  |  |  |
| 25．我觉得一切都很好，也不会发生什么不幸 |  |  |  |  |
| 26．我手脚发抖打颤 |  |  |  |  |
| 27．我因为头痛、颈痛和背痛而苦恼 |  |  |  |  |
| 28．我感觉容易衰弱和疲乏 |  |  |  |  |
| 29．我觉得心平气和，并且容易安静坐着 |  |  |  |  |
| 30．我觉得心跳得很快 |  |  |  |  |
| 31．我因为一阵阵头晕而苦恼 |  |  |  |  |
| 32．我有晕倒发作，或觉得要晕倒似的 |  |  |  |  |
| 33．我吸气呼气都感到很容易 |  |  |  |  |
| 34．我的手脚麻木和刺痛 |  |  |  |  |
| 35．我因为胃痛和消化不良而苦恼 |  |  |  |  |
| 36．我常常要小便 |  |  |  |  |
| 37．我的手脚常常是干燥温暖的 |  |  |  |  |
| 38．我脸红发热 |  |  |  |  |
| 39．我容易入睡并且一夜睡得很好 |  |  |  |  |
| 40．我做恶梦 |  |  |  |  |
