# Supplementary material for: Associations of Physical Activity, Screen Time with Depression, Anxiety and Sleep Quality among Chinese College Freshmen
Source: PLoS One. 2014 Jun 25;9(6):e100914. doi: 10.1371/journal.pone.0100914 (PMC4071010; doi:10.1371/journal.pone.0100914)
Supplement: Questionnaire S2 — Questionnaire in English. (DOC) [file pone.0100914.s002.doc]

**Questionnaire**

1. Your Gender ①Male ②Female

2. Your Birth Date Year____Month_____Day___

3.Your Height ________cm，Weight________kg

4. Do you have any chronic diseases such as hypertension, heart disease, asthma?

①No ②Yes

5. What is the highest level of education completed by your father?

①college or higher(>12years) ②high school、technical secondary school or vocational school (10-12years) ③junior high school (7-9years) ④primary school or lower (≤6years)

6.What is the highest level of education completed by your mother?

①college or higher(>12years) ②high school、technical secondary school or vocational school (10-12years) ③junior high school (7-9years) ④primary school or lower (≤6years)

7. How often do you sport and/or vigorous free play each week with 30 minutes at least per day?

① every day ② 5-6 days/week ③ 3-4days/week ④ 1-2days/week ⑤ <1day/week

8. **From Monday to Friday**，How many hours per day do you spend on computer, including internet use, watching TV/video programs and playing games _____hour(s)/day

9. **From Saturday to Sunday**，How many hours per day do you spend on computer, including internet use, watching TV/video programs and playing games _____hour(s)/day

**Read each sentence carefully. For each statement, select the response that best corresponds to your feelings by using “√” 。**

10. **During the past one month**, how often have you had trouble sleeping because you . .：

| **Problems** | **Not during the past month** | **Less than once a week** | **Once or twice a week** | **Three or more times a week** |
| --- | --- | --- | --- | --- |
| A Cannot get to sleep within 30 minutes |  |  |  |  |
| B Wake up in the middle of the night or early morning |  |  |  |  |
| C Have to get up to use the bathroom |  |  |  |  |
| D Cannot breathe comfortably |  |  |  |  |
| E Cough or snore loudly |  |  |  |  |
| F Feel too cold |  |  |  |  |
| G Feel too hot |  |  |  |  |
| H Had bad dreams |  |  |  |  |
| I Have pain |  |  |  |  |
| J Other reason(s) |  |  |  |  |

.11. During the past month, how would you rate your sleep quality overall?

①Very good ②Fairly good ③Fairly bad ④Very bad

12. During the past month, how often have you taken medicine to help you sleep (prescribed or

"over the counter")?

①Not during the past month ②Less than once a week ③Once or twice a week

④ Three or more times a week

13. During the past month, how often have you had trouble staying awake while driving, eating

meals, or engaging in social activity?

①Not during the past month ②Less than once a week ③Once or twice a week

④Three or more times a week

14. During the past month, how much of a problem has it been for you to keep up enough

enthusiasm to get things done?

①No problem at all ②only a very slight problem ③somewhat of a problem

④a very big problem

**------------------------------------------------------------------------------**

**I. Read each sentence carefully. For each statement, select the response that best corresponds to how often you have felt that way in the last 1 week.**

| **Have you experienced situations listed below in the last 1 week？** | **A little of the time** | **Some of the time** | **Good part of the time** | **Most of the time** |
| --- | --- | --- | --- | --- |
| 1．I feel down-hearted and blue |  |  |  |  |
| 2．Morning is when I feel the best |  |  |  |  |
| 3．I have crying spells or feel like it |  |  |  |  |
| 4．I have trouble sleeping at night |  |  |  |  |
| 5．I eat as much as I used to |  |  |  |  |
| 6．I still enjoy sex |  |  |  |  |
| 7．I notice that I am losing weight |  |  |  |  |
| 8．I have trouble with constipation |  |  |  |  |
| 9．My heart beats faster than usual |  |  |  |  |
| 10．I get tired for no reason |  |  |  |  |
| 11．My mind is as clear as it used to be |  |  |  |  |
| 12．I find it easy to do the things I used to |  |  |  |  |
| 13．I am restless and can't keep still |  |  |  |  |
| 14．I feel hopeful about the future |  |  |  |  |
| 15．I am more irritable than usual |  |  |  |  |
| 16．I find it easy to make decisions |  |  |  |  |
| 17．I feel that I am useful and needed |  |  |  |  |
| 18．My life is pretty full |  |  |  |  |
| 19．I feel that others would be better off if I were dead |  |  |  |  |
| 20．I still enjoy the things I used to do |  |  |  |  |
| 21．I feel more nervous and anxious than usual |  |  |  |  |
| 22．I feel afraid for no reason at all |  |  |  |  |
| 23．I get upset easily or feel panicky |  |  |  |  |
| 24．I feel like I’m falling apart and going to pieces. |  |  |  |  |
| 25．I feel that everything is all right and nothing bad will happen |  |  |  |  |
| 26．My arms and legs shake and tremble. |  |  |  |  |
| 27．I am bothered by headaches neck and back pain. |  |  |  |  |
| 28．I feel weak and get tired easily |  |  |  |  |
| 29．I feel calm and can sit still easily. |  |  |  |  |
| 30．I can feel my heart beating fast. |  |  |  |  |
| 31．I am bothered by dizzy spells. |  |  |  |  |
| 32．I have fainting spells or feel like it. |  |  |  |  |
| 33．I can breathe in and out easily. |  |  |  |  |
| 34．I get numbness and tingling in my fingers and toes. |  |  |  |  |
| 35．I am bothered by stomach aches or indigestion. |  |  |  |  |
| 36．I have to empty my bladder often. |  |  |  |  |
| 37．My hands are usually dry and warm. |  |  |  |  |
| 38．My face gets hot and blushes. |  |  |  |  |
| 39．.I fall asleep easily and get a good night’s rest. |  |  |  |  |
| 40．I have nightmares. |  |  |  |  |
